# Sporadic Infantile Epileptic Encephalopathy Caused by Mutations in *PCDH19* Resembles Dravet Syndrome but Mainly Affects Females

**DOI:** 10.1371/journal.pgen.1000381

**Published:** 2009-02-13

**Authors:** Christel Depienne, Delphine Bouteiller, Boris Keren, Emmanuel Cheuret, Karine Poirier, Oriane Trouillard, Baya Benyahia, Chloé Quelin, Wassila Carpentier, Sophie Julia, Alexandra Afenjar, Agnès Gautier, François Rivier, Sophie Meyer, Patrick Berquin, Marie Hélias, Isabelle Py, Serge Rivera, Nadia Bahi-Buisson, Isabelle Gourfinkel-An, Cécile Cazeneuve, Merle Ruberg, Alexis Brice, Rima Nabbout, Eric LeGuern

**Affiliations:** 1 AP-HP, Département de Génétique et Cytogénétique, Fédération de Génétique, Hôpital de la Salpêtrière, Paris, France; 2 INSERM U975 (Ex-U679), Paris, France; 3 Université Pierre et Marie Curie-Paris 6, CNRS, UMR-S975, Paris, France; 4 Service de Neurologie Pédiatrique, Hôpital des Enfants, Centre Hospitalier Universitaire de Toulouse, Toulouse, France; 5 Institut Cochin, Inserm U567, UMR 8104, Université René Descartes, Paris, France; 6 Plate-forme Post-Génomique P3S, UPMC, Faculté de Médecine, Paris, France; 7 Service de Neuropédiatrie, Hôpital Trousseau, Paris, France; 8 Service de Neuropédiatrie, CHU Nantes, Nantes, France; 9 Service de Neuropédiatrie - Hôpital Gui de Chauliac, CHU de Montpellier, Montpellier, France; 10 Service de Neuropédiatrie, Hôpital de Bordeaux, Bordeaux, France; 11 Service de Neuropédiatrie, CHU Hôpital Nord Amiens, Amiens, France; 12 ITEP de Champthierry et ASPEC, Mortagne-au-Perche, France; 13 Service de Pédiatrie, Centre Hospitalier de Cholet, Cholet, France; 14 Service de Pédiatrie, Hôpital de Bayonne, Bayonne, France; 15 Département de Neuropédiatrie, AP-HP, Hôpital Necker-Enfants Malades, Paris-Descartes, Paris, France; 16 Pôle d'Epileptologie, Hôpital de la Salpêtrière, Paris, France; 17 Centre de Référence Épilepsies Rares, Paris, France; University of Michigan, United States of America

## Abstract

Dravet syndrome (DS) is a genetically determined epileptic encephalopathy mainly caused by de novo mutations in the *SCN1A* gene. Since 2003, we have performed molecular analyses in a large series of patients with DS, 27% of whom were negative for mutations or rearrangements in *SCN1A*. In order to identify new genes responsible for the disorder in the *SCN1A*-negative patients, 41 probands were screened for micro-rearrangements with Illumina high-density SNP microarrays. A hemizygous deletion on chromosome Xq22.1, encompassing the *PCDH19* gene, was found in one male patient. To confirm that *PCDH19* is responsible for a Dravet-like syndrome, we sequenced its coding region in 73 additional *SCN1A*-negative patients. Nine different point mutations (four missense and five truncating mutations) were identified in 11 unrelated female patients. In addition, we demonstrated that the fibroblasts of our male patient were mosaic for the *PCDH19* deletion. Patients with *PCDH19* and *SCN1A* mutations had very similar clinical features including the association of early febrile and afebrile seizures, seizures occurring in clusters, developmental and language delays, behavioural disturbances, and cognitive regression. There were, however, slight but constant differences in the evolution of the patients, including fewer polymorphic seizures (in particular rare myoclonic jerks and atypical absences) in those with *PCDH19* mutations. These results suggest that *PCDH19* plays a major role in epileptic encephalopathies, with a clinical spectrum overlapping that of DS. This disorder mainly affects females. The identification of an affected mosaic male strongly supports the hypothesis that cellular interference is the pathogenic mechanism.

## Author Summary

Severe epilepsies associated with cognitive impairment in children are multifarious and most affected patients are sporadic cases. Thus, there is a challenge to identify which of these epilepsies are genetically determined, since their sporadic status excludes the use of classical genetic approaches. We have used microarrays, which are new technological tools to investigate the whole genome of an individual, to search for small genomic abnormalities and identify novel genes in 41 patients with a clinically well-characterized severe infantile epileptic disorder called Dravet syndrome. We have identified *PCDH19*, a new gene on chromosome X, which was recently found in a familial epileptic syndrome known as female-limited epilepsy and cognitive impairment. This gene was mutated in 12 out of 74 patients with clinical features compatible with Dravet syndrome. Eleven of these patients were females. The single male with a *PCDH19* deficiency was mosaic in his skin; i.e., some of his cells express *PCDH19* and others do not. This finding suggests that a new pathogenic mechanism—cellular interference—is associated with an unusual X-linked mode of inheritance in which females are more frequently affected than males.

## Introduction

Epileptic encephalopathies are a group of rare disorders in which impairment of cognitive, behavioural and other brain functions is caused by the same underlying disease process. This heterogeneous group of disorders has multiple aetiologies such as symptomatic brain lesions, metabolic causes and diverse genetic syndromes. Much progress has been made in the past few years in the identification of genes responsible for genetic infantile epileptic encephalopathies. Among the genetic syndromes that have been characterized are: Dravet syndrome (DS), also called severe myoclonic epilepsy of infancy (SMEI, MIM# 607208) [1], CDKL5/STK9 Rett-like epileptic encephalopathy [2],[3], ARX-related epileptic encephalopathies [4], SRPX2-related rolandic epilepsy associated with oral and speech dyspraxia and mental retardation [5], and very recently, female-limited epilepsy and cognitive impairment (EFMR) associated with mutations in *PCDH19*, the gene encoding the protocadherin 19 on the X chromosome [6].

Dravet syndrome is characterized by the occurrence of generalized or unilateral clonic or tonic–clonic seizures, usually triggered by fever, in the first year of life of a previously normal infant. Later on, other types of seizures occur, including myoclonus, atypical absences and partial seizures [7]. Development is progressively delayed starting from the second year. Susceptibility to febrile seizures persists over time, and *status epilepticus* is frequent. Epilepsy generally persists despite appropriate anti-epileptic therapy (polytherapy including sodium valproate, clobazam or topiramate and stiripentol). Children with DS typically have poorly developed language and motor skills, learning disabilities and variable degrees of mental retardation [8]. They are usually sporadic cases; however, sib pairs with SMEI, or patients with a family history of epilepsy, have occasionally been reported [9].

Heterozygous de novo mutations in *SCN1A*, the gene encoding the voltage-gated neuronal sodium channel alpha 1 subunit (Nav1.1), are a major cause of DS [1]. All types of mutations [10] and rearrangements [11]–[15] in *SCN1A* have been observed in SMEI patients. However, no point mutations or rearrangements have been found in a fraction of patients, now estimated to 20–25% [15]–[18], strongly suggesting that DS is a genetically heterogeneous disorder.

Our aim was to identify the gene(s) involved in *SCN1A*-negative patients with Dravet syndrome. Most of our patients were isolated, excluding the use of classical genetic approaches. Our hypothesis was that genomic micro-rearrangements, which are increasingly identified as causes of human genetic disorders, might be found in a subset of the *SCN1A*-negative patients with DS, thus identifying new causal genes.

In this study, we have searched for genomic rearrangements in 41 *SCN1A*-negative patients using high-density SNP microarrays (Illumina, 370K). Genes located in the rearrangements were then considered to be candidate genes and were analysed for point mutations by direct sequencing in the remaining negative patients with DS.

## Results

### Microarray-Based Identification of a De Novo Deletion on Chromosome Xq22.1 Encompassing *PCDH19*

**Figure 1:**
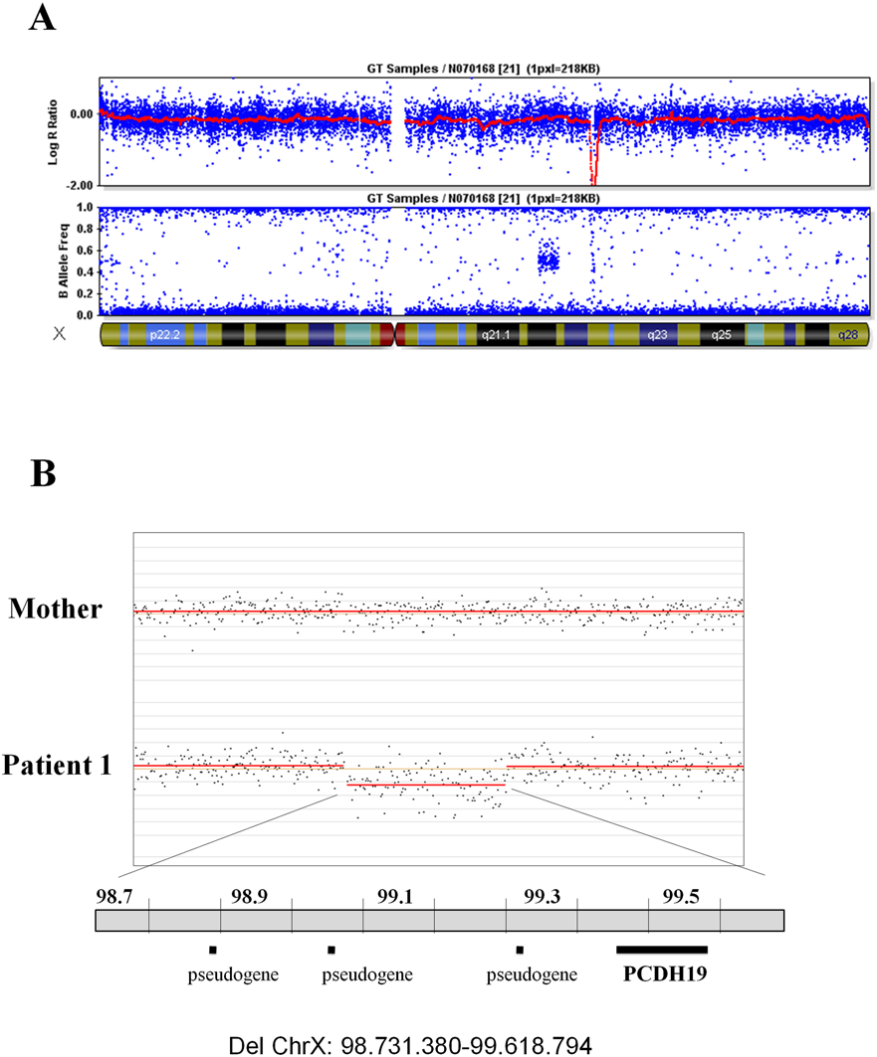
Identification of a deletion encompassing *PCDH19* in a male patient. A) Identification of a hemizygous Xq22.1 deletion with a 370 K SNP microarray (Illumina): Y-axes represent Log R ratio (above) and B allele frequency (below); the X-axis indicates the position on the X chromosome. The red line (log R ratio profile) corresponds to the median smoothing series (Beadstudio). B) Analysis of the patient and his mother with CGH microarrays (Nimblegen), showing that the deletion occurred de novo. Indicated genomic positions are based upon the Ensembl Genome Browser. Black horizontal bars (below) represent the gene (*PCDH19*) and pseudogenes comprised in the deleted region.

An initial series of 41 probands (18 females and 23 males), referred for genetic analysis of Dravet syndrome but negative for point mutation and intragenic rearrangement of *SCN1A* [15], was screened for genomic rearrangement using Illumina 370CNV microarrays. A hemizygous deletion on chromosome Xq22.1 was identified in a male patient (patient 1 from family 1). This deletion spanned approximately 1 Mb and encompassed a single gene, *PCDH19* (Figure 1A). A duplication of the same region was previously reported in one of 776 healthy controls (506 unrelated healthy individuals from Northern Germany and 270 HapMap subjects) [19], but no deletions in healthy individuals have been recorded in the database of genomic variants. Patient 1 and his mother were then analyzed with high-resolution CGH arrays (Nimblegen). This analysis confirmed that the deletion spans 890 Kb, between genomic positions g.98731380 and g.99618794 on chromosome X, and showed that it has occurred de novo since it was not found in the mother of the patient (Figure 1B).

### Identification of Additional Patients with Point Mutations in the Coding Sequence of *PCDH19* by Direct Sequencing

*PCDH19* encodes protocadherin 19, a transmembrane protein of the cadherin family of calcium-dependent cell–cell adhesion molecules, which is strongly expressed in the central nervous system. In the postnatal brain, protocadherins might be involved in the modulation of synaptic transmission and the generation of specific synaptic connections [20]. *PCDH19* was therefore an attractive candidate gene for epilepsies and mental retardation. To test whether a *PCDH19* deficiency might be implicated in some epileptic encephalopathies resembling Dravet syndrome, we sequenced the coding region of this gene in 73 *SCN1A*-negative probands (the remaining 40 patients of the initial series plus 33 additional patients, for a total of 45 females and 28 males).

**Figure 2:**
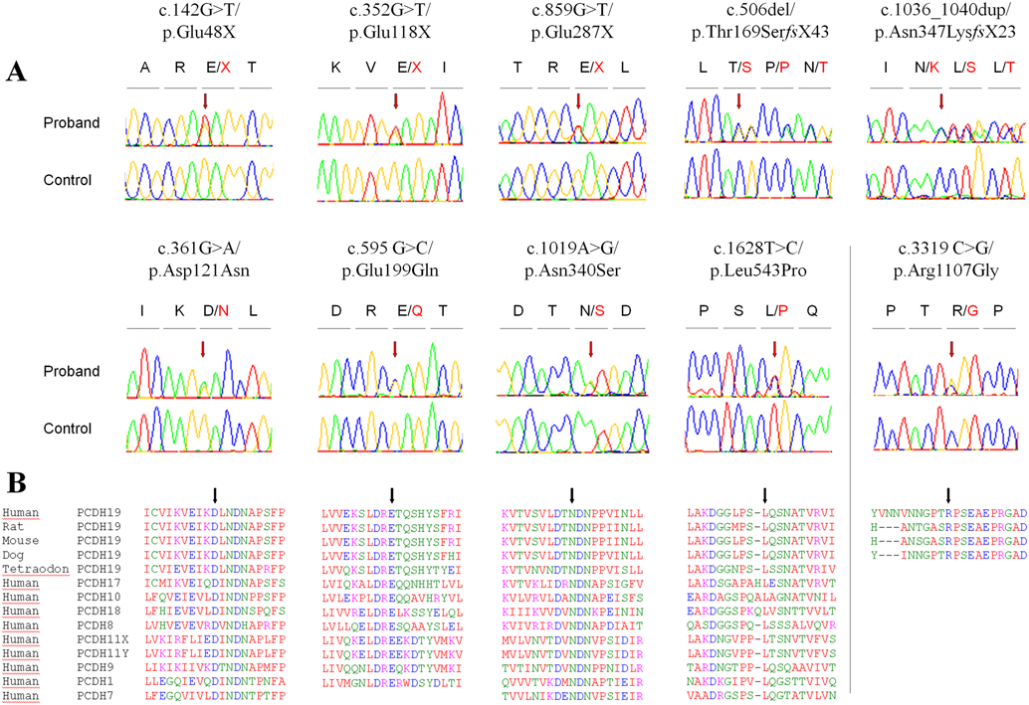
Detection of 9 different point mutations of *PCDH19* in 11 female patients by direct sequencing. A) Sequence electropherograms of the mutations and the missense variant (c.3319C>G/p.Arg1107Gly) identified in association with the c.859G>T/p.Glu287X nonsense mutation. The mutation nomenclature is based on the *PCDH19* transcript reference EF676096. Nucleotides are numbered according to the cDNA with +1 corresponding to the A of the ATG translation initiation codon in the reference sequence, according to the journal guidelines (www.hgvs.org/mutnomen). B) Alignment of the regions surrounding the mutations (indicated by an arrow) in orthologous and paralogous proteins, showing the high conservation of each affected amino-acid in vertebrates and in the delta protocadherin paralogous genes.

Ten different variants were identified in 11 unrelated female probands at the heterozygous state (Figure 2A). All but one were located in exon 1: three were nonsense mutations (c.142G>T/p.Glu48X, c.352G>T/p.Glu118X, c.859G>T/p.Glu287X), two were small deletions and insertions creating a frameshift (c.506delC/p.Thr169SerfsX43 and c.1036_1040dup/p.Asn347Lys*fs*X23) and the remaining five were missense mutations (c.361G>A/p.Asp121Asn, c.595 G>C/p.Glu199Gln, c.1019A>G/p.Asn340Ser, c.1628 T>C/p.Leu543Pro and c.3319 C>G/p.Arg1107Gly). Glu48X was present in two affected sisters of family 2; Glu118X was identified in an isolated patient (family 3) and Glu287X was found independently in a patient with family history of epilepsy and mental retardation (family 4) and in an isolated patient (family 5). Interestingly, the c.3319 C>G/p.Arg1107Gly missense variant, located in exon 6, was associated with the p.Glu287X mutation in the proband of family 5. In family 6, cytosine 506 (c.506delC) was deleted in a patient whose parents were unaffected, but whose female cousin also had epilepsy and moderate mental retardation. The 5-bp duplication (c.1036_1040dup) was present in the index case of family 7. The p.Asp121Asn mutation was identified in the index case of family 8, who had a sister with epilepsy and psychotic disturbances. Finally, p.Glu199Gln, p.Asn340Ser and p.Leu543Pro variants were identified in the 4 remaining isolated patients (families 9 to 12); Asn340Ser was found in two independent patients (families 10 and 11). These 4 missense variants (p.Asp121Asn, p.Glu199Gln, p.Asn340Ser and p.Leu543Pro) all affected amino-acids in the extracellular domain of protocadherin 19, which are highly conserved in orthologs and in paralogs of *PCDH19* in the delta protocadherin family (Figure 2B). Interestingly, p.Arg1107Gly, associated with the de novo Glu287X mutation in the proband of family 5, affected a residue of the protein that is conserved in mammalian orthologs, but not in other species or in paralogs (Figure 2B). To confirm that the variants are pathogenic, we screened 180 healthy Caucasians. Only Arg1107Gly was found in a healthy female individual and was thus considered to be a rare polymorphism. None of the other variants was found in the control population, confirming that they are causal mutations.

**Figure 3:**
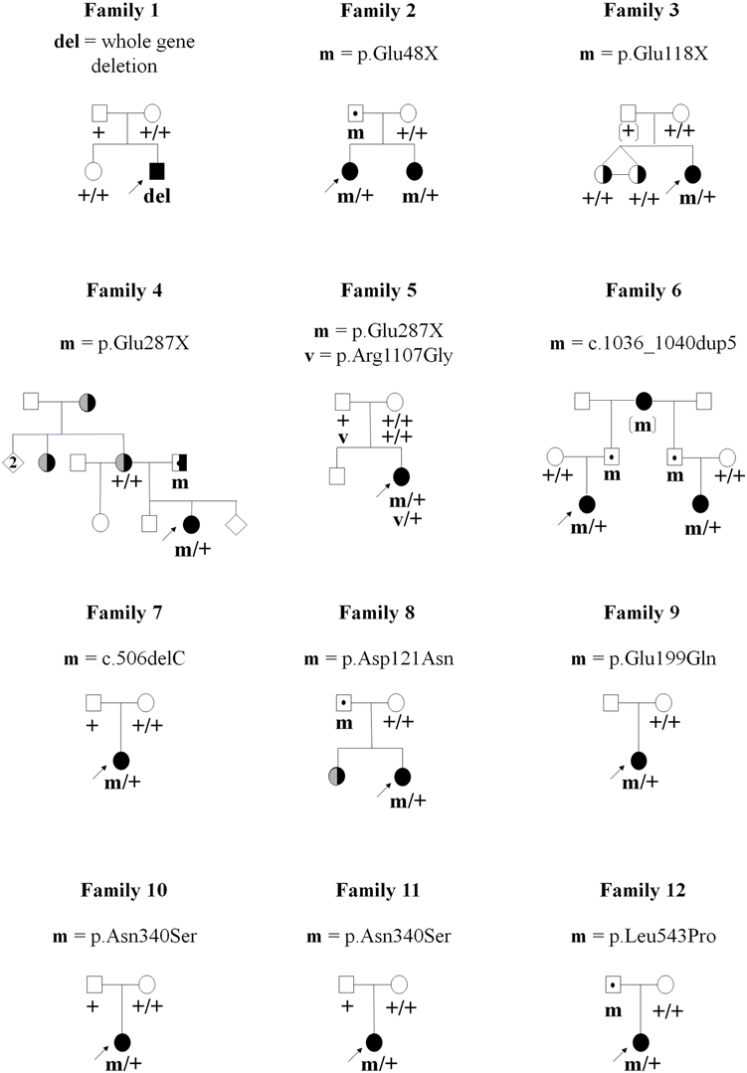
Pedigrees of the families and segregation analysis of the *PCDH19* deletion and point mutations. del/+, m/+ or v/+ denote individuals heterozygous for the deletion, mutation or variant, respectively; +/+ denotes individuals carrying homozygous wild-type alleles. Squares represent males, circles females; filled black symbols: patients diagnosed as having Dravet syndrome; right black half: Cognitive delay or impairment; left grey half: adolescence-onset idiopathic epilepsy. Dots in the middle of the squares indicate unaffected mutation carriers. The arrows indicate the index cases.

The parents and relatives of *PCDH19*-positive patients were also analysed when possible (Figure 3). The p.Glu48X mutation, found in two affected sibs in family 2, was inherited from their asymptomatic father. Likewise, the c.1036_1040dup5, p.Asp121Asn and p.Leu543Pro mutations were inherited from the healthy fathers of the index cases in families 6, 8 and 12. In family 6, the 5-bp duplication was inherited from the paternal grandmother who also had epilepsy and cognitive impairment, and transmitted to the half-brother of the father and his affected daughter (i.e. the index case's cousin, Figure 3). In family 4, the mother of the proband had mental retardation associated with adult-onset epilepsy, a clinical feature also present in the maternal grandmother and maternal aunt; the proband's father also presented with moderate mental retardation but without epilepsy. The Glu287X mutation in this family was also inherited from the father. In contrast, in families 5, 7, 10 and 11, the mutations (p.Glu287X, c.506delC and p.Asn340Ser, respectively) occurred de novo in the index cases, since they were not found in either parent. Interestingly, Arg1107Gly was inherited from the asymptomatic father in family 5. In family 4, only the mother and sisters of the index case were available for genetic analyses. Both sisters, who were monozygous twins, had mild psychomotor and cognitive impairment but never had seizures. Neither the mother nor the sisters had the p.Glu287X mutation. Analysis of the haplotypes in Xq22.1 (PCDH19 locus) with microsatellite markers confirmed that the three sisters (the two twins and the affected proband) received the same X chromosome from their father, with and without the p.Glu287X mutation, which indicates that the mutation also occurred de novo in this family. Finally, in family 9, the mother did not have the p.Glu199Gln mutation but the father remained unavailable for genetic analyses.

### FISH Shows Somatic Mosaïcism in the Male Patient with the *PCDH19* Deletion

Recently, *PCDH19* mutations were shown to cause epilepsy and mental retardation limited to females (EFMR), a familial disorder associating childhood-onset epilepsy and a variable degree of cognitive impairment with an unusual mode of inheritance: this X-linked disorder is found in females with heterozygous mutations but not in males with hemizygous mutations [6]. How, then, can we explain the affected male in our series with a deletion of the entire *PCDH19*? Random X-inactivation in mutated females normally leads to tissue mosaicism in which two cell populations, one expressing normal *PCDH19* and the other expressing the mutated allele, co-exist. To explain why only females are affected, it might be hypothesized that the co-existence of *PCDH19*-positive and *PCDH19*-negative cells would be pathogenic whereas homogeneous cell populations (*PCDH19*-positive in normal individuals but *PCDH19*-negative in mutated males) would not [6]. A mechanism of this type was previously termed “cellular interference” [21]. Two cell populations would also be found in mosaic males, who, according to this hypothesis, would be affected like mutated females.

**Figure 4:**
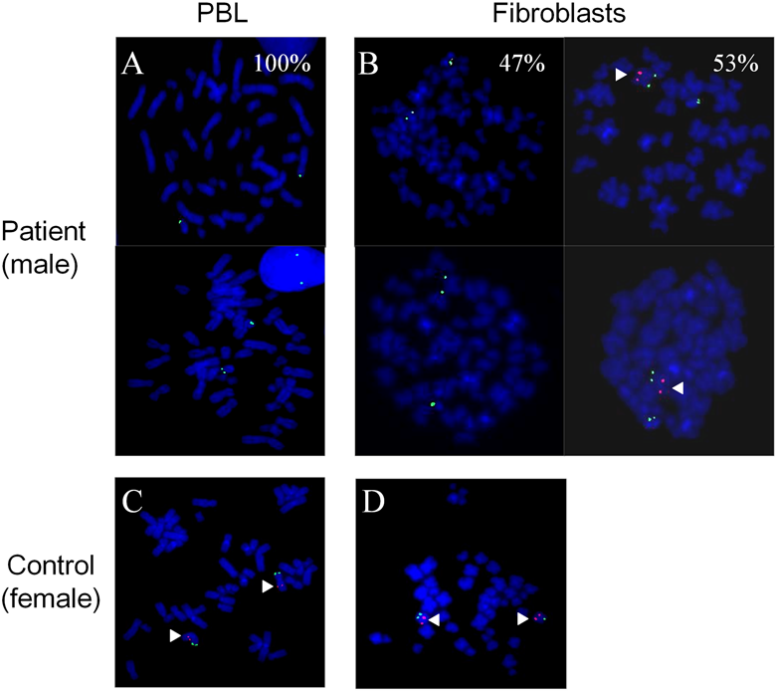
FISH analysis of the *PCDH19* deletion in the male patient showing somatic mosaicism in fibroblasts. (A) Absence of the specific Xq22.1 probe site on metaphase chromosomes in peripheral blood lymphocytes (PBL); (B) In fibroblasts, presence of one hybridization spot in 53% of the cells and absence of signal in the remaining 47%; C) and D) FISH analysis on PBL (C) and fibroblasts (D) of a female control. *PCDH19*-specific signals (red) are indicated by arrowheads. Magnification ×1000.

To test whether our male patient was mosaic for the *PCDH19* gene deletion, we compared peripheral blood lymphocytes (PBL) and cultured fibroblasts from the patient by FISH with a probe specific to the *PCDH19* genomic region. Although no signal corresponding to *PCDH19* was detectable in PBL, a normal *PCDH19* allele was found in 53% of the fibroblasts (Figure 4), confirming that the patient was mosaic, in his skin, for the *PCDH19* deletion. This result confirms that mutations in *PCDH19* can be responsible, in mosaic males, for epileptic encephalopathy phenotypes that are usually limited to females, and strongly supports the hypothesis that cellular interference is the main pathogenic mechanism of the disease.

### Clinical Features of the Patients with *PCDH19* Mutations or Deletions

The clinical features of the male patient with the *PCDH19* deletion and the female patients with *PCDH19* point mutations are summarized in Table 1. These patients fulfil the main criteria for DS (see material and methods' section), with a mean age of seizures onset of 9.5 months (ranging from 7.5 to 12 months). Nevertheless, contrary to *SCN1A*-positive patients (SCN1A-DS), myoclonic jerks, atypical absences, and photosensitivity were unfrequent in *PCDH19*-positive patients (PCDH19-DS) (3, 3 and 1 patients out of 13, respectively). Only 6 patients presented *status epilepticus*. The mental delay was mild in 6 patients, moderate in 4 and only 3 patients presented with severe delay. Although much delayed, the language was present in all patients, with 12 out of 13 able to formulate short sentences.

**Table 1:** Clinical characteristics of patients with *PCDH19* mutations.

Family number	1	2	2*	3	4	5	6	7	8	9	10	11	12
**Patient**	N 07 0168	N 06 1257	N 06 1258	N 06 1358	N 07 0627	N 07 0971	18050_31839	N 06 0730	N 07 0661	N 07 1000	N 06 1016	N 06 0991	N 05 1157
**Sex**	M	F	F	F	F	F	F	F	F	F	F	F	F
**Present age (years)**	7	6	3	7	3	13	10	12	2,5	18	3,5	6	8
**PMD previous to seizures onset**	Nl	Nl	Nl	Nl	?	Nl	Nl	Nl	?	Nl	Nl	Nl	Nl
**Age at seizures onset (months)**	12	9	11	11	10	7.5	11	9	9	?	9	8	8
**Type of seizures at onset**	F, GTC, prolonged, repetitive	Focal	Unilat	F, GTC, prolonged, repetitive	GTC	F, unilat	F, unilat	GTC	Unilat	F,unilat	F, GTC	Partial	F,unilat
**Presence of febrile seizures (FS)**	+(>50%)	+	+	+(>50%)	+(>50%)	+(>50%)	+(50%)	+(>50%)	+(>50%)	+(>50%)	+(>50%)	+(>50%)	+(>50%)
**Unilateral (or hemiclonic) seizures**	+	+	−	−	+	+	+	+	+	+	+	+	+
**Other seizure types**													
- Partial	+	+	+	−	+	+	+	+	+	+	+	+	+
- GTC	+	+	+	+	+	+	+	+	+	+	+	+	+
- Myoclonic jerks	+	−	−	−	−	−	+	−	−	−	−	−	−
- Absences	?	+	+	?	−	−	+	−	−	−	−	−	−
**Repetitive seizures (in clusters)**	+	+	+	+	+	+	+	+	+	+	−	+	+
***Status epilepticus***	+	+	−	−	−	+	−	−	+	+	+	−	−
**Photosensitivity**	−	−	−	−	−	−	−	−	−		−	+	−
**Persistance of seizures in spite of treatment**	+(F)	+	−	+	+	+	+	−	+	+	−	+	+
**Mental retardation**	moderate/severe	mild	mild	moderate	moderate/severe	severe	mild	moderate	mild	moderate	mild	mild	moderate
**Language delay**	+(W-S)	+(W-S)	−	+(W-S)	+(abs)	+(W-S)	+(W-S)	+(W-S)	+(W)	+(W-S)	+(W-S)	+(W-S)	+(W-S)
**Behavioral disturbances**	+	+	−	+	+	+	+	?	?	−	−	+	+
**Autistic features**	+	−	−	+	−	−	−	−	−	−	−	−	−
**Motor delay**	+	+(mild)	+(mild)	+	+(Hypotonia)	+	+(mild)	−	+	−	−	+	+
**Ataxia**	+	+	+(mild)	+	+	+	−	?	−	−	−	+	+
**Other clinical features**	−	−	−	Tall stature	Hyperlaxity	−	−	−	−	−	−	−	−
**Treatment (AED)**													
- Sodium Valproate	+	+	+	+	+	+	+	+	+	+	+	+	
- Clobazam	+	+		+	+		+	+			+	+	+
- Clonazepam	+			+	+	+			+	+			
- Topiramate	+					+		+	+	+		+	+
- Stiripentol	+						+				+		+
- Lamotrigine		+	+	+									

2*: patient N 06 1258 is the sister of patient N 06 1257 (index case); PMD = psychomotor development, Nl = normal, F = febrile, unit = unilateral, GTC = generalized tonic-clonic, W-S = words-sentences, abs = absent, AED = anti epileptic drugs.

## Discussion

In this study, we used SNP microarrays to search for microrearrangements in patients with clinical features suggestive of Dravet syndrome but without mutations in *SCN1A* in order to identify new causative genes. The identification of a de novo hemizygous deletion of *PCDH19*, encoding protocadherin 19, in a male patient led us to screen the coding region of this gene in the remaining patients. Eleven unrelated probands with point mutations in *PCDH19*, all females, were found. While this study was ongoing, *PCDH19* was reported to be the causative gene for female-limited epilepsy and cognitive impairment (EFMR), a disorder characterized by seizure onset in infancy or early childhood and cognitive impairment, which is found only in females in multi-generational families [6]. Since all of our patients with point mutations in *PCDH19* were females as previously reported, we investigated the possibility that the male patient in whom the gene was deleted might be mosaic for the deletion. FISH analysis confirmed this latter hypothesis.

The thirteen patients with *PCDH19* mutation or deletion (12 probands and one sib, family 2) all fulfilled the main criteria for DS and were all negative for mutation or rearrangement in *SCN1A* after direct sequencing and multiplex ligation-dependent probe amplification (MLPA) [15]. The proportion of PCDH19-DS probands in our series of *SCN1A*-negative patients was 16% (12/74), or even 25% (11/45) if only female patients were included in the calculation. Considering that approximately 25% of all patients with DS are *SCN1A*-negative [15], *PCDH19* might overall account for 5% of DS patients. PCDH19-DS patients and SCN1A-DS patients have many features in common including: normal psychomotor development before seizures onset, early onset of seizures (before age one year), association of febrile and afebrile seizures, with a high susceptibility of the seizures to fever for all 13 patients, occurrence of hemiclonic or unilateral seizures (11/13), and association of generalized tonic-clonic and focal seizures (12/13), a high proportion of seizures occurring in clusters (12/13), prolonged seizures, a proportion of which lead to *status epilepticus*, secondary progressive appearance of mental and motor regression and language delay, accompanied, in some cases, with ataxia (Table 1).

However, PCDH19-DS patients slightly differ on average from the classical pattern reported in SCN1A-DS. PCDH19-DS patients were slightly older at onset than SCN1A-DS patients (9.5 months, with a range from 7.5 to 12 months, versus 6.3 months, calculated from our series of SCN1A-positive DS patients, p<0.0001) [15]. Less than half (6/13) of the PCDH19-DS patients had *status epilepticus* although this is a highly frequent feature in SCN1A-DS (93/113, p<0.007). Photosensitivity, frequently reported in SCN1A-DS, was exceptional in PCDH19-DS and was reported in only one patient but the difference with SCN1A-DS in our series of patients was however not significant. Seizures were, on average, less intractable than in SCN1A-DS, and patients above six years of age (9/12 patients) had less than 4 seizures a year with one patient who was free of seizures at the time of the study. Although all patients were on tri- or poly-therapy, seizures were relatively well-controlled, a situation rarely achieved in SCN1A-DS. Intellectual and language delay were constant but were less severe than the classical outcome of SCN1A-DS [8] (mostly with important speech and mental delay) although the difference was not significant. Finally, myoclonic jerks and atypical absences were present in only 2 and 3 patients, respectively, whereas they are frequent features in SCN1A-DS (myoclonic jerks: 55/110, p<0.018; atypical absences: 92/108, p<0.0001). Patients with SMEI but without myoclonia have been previously referred as SMEB (borderline severe myoclonic epilepsy in infancy) [22], but SMEI and SMEB are currently grouped together under the term DS. In addition, the same types of mutation, and even the same mutations, are found in patients with DS and patients with other infantile epileptic encephalopathies (such as cryptogenic generalized or focal epilepsies), which has extended the clinical spectrum of *SCN1A* and the definition of DS [15]. Therefore, in individuals, these divergent clinical characteristics are not sufficient to distinguish between patients with *SCN1A* or *PCDH19* mutations, and the two clinical spectrums largely overlap. They can be useful, however, to prioritize molecular diagnosis although they must be first confirmed on larger series.

Mutations in *PCDH19* were recently reported to cause EFMR, which also associates mental retardation and epilepsy exclusively in females. EFMR was differentiated from DS by the authors on both clinical and genetic grounds [6],[23]. The clinical features of EFMR, unlike those of DS, are highly variable, even in members of the same family: onset of seizures is between 6 and 36 months, the patients present with a combination of febrile and afebrile seizures of various types and a variable degree of psychomotor delay and cognitive impairment, ranging from mild to severe mental retardation [23]. Dibbens *et al.* reported *PCDH19* mutations in six large families and one small family with two affected sib pairs [6]. All the patients were familial cases that were, for the most part, already adults at the time of examination, and appeared socially integrated in that most of them were married and had children. In the present study, on the contrary, the patients were essentially young, had a severe epileptic encephalopathy, and 8 of the 12 were isolated cases. In 6 patients out of 11 in whom inheritance could be assessed, the mutation occurred de novo. In the 5 remaining patients, the mutation was inherited from fathers who were healthy, had no cognitive impairment, and never had febrile seizures or epilepsy (families 2, 6, 8 and 12), or had mild mental retardation but no epilepsy (family 4). The global clinical pictures of PCDH19-DS and EFMR appear therefore to differ. The difference in the phenotypes might be due to the modes of recruitment (familial *versus* sporadic cases). It might also be hypothesized, that patients with PCDH19-DS have a better final outcome than in those with SCN1A-DS despite the severity of their disease in childhood and that the two disorders are different clinical expressions of the same disease. Both hypotheses are not mutually exclusive. Interestingly, the variability in the severity of epilepsy and cognitive impairment in EFMR is reminiscent of what is observed in GEFS+ families (generalized epilepsy with febrile seizures plus, # MIM# 604233), an autosomal dominant condition that also associates febrile seizures with epilepsy of variable types and severity, and which is associated in ∼10–15% of the families with missense mutations in *SCN1A* [10]. Although patients with GEFS+ are usually responsive to treatment and generally have a benign outcome, some family members may be more severely affected, and even present with DS. The clinical spectrum of *PCDH19* mutations could be as broad as the spectrum of GEFS+. Random X inactivation could contribute to this variability by generating variable proportions of mutated to normal cells in the brains of the mutated females.

**Figure 5:**
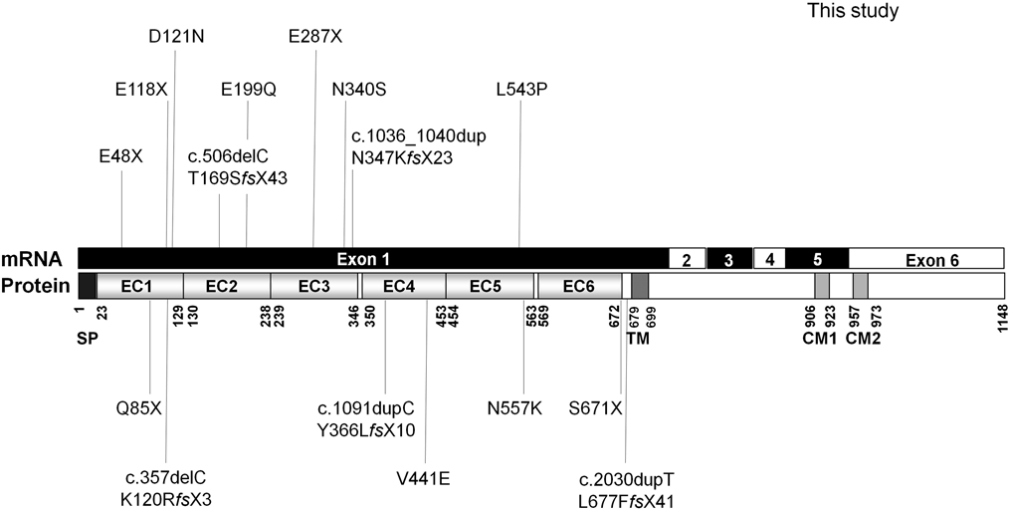
Schematic representation of the point mutations identified in the *PCDH19* gene. Above: mutations identified in this study; Below: mutations identified by Dibbens et al. (2008). SP: signal peptide; EC: extracellular cadherin domain; TM: transmembrane domain; CM1 and CM2: cytoplasmic domains 1 and 2.

Although the mutations in EFMR families and in PCDH19-DS patients are distinct, the spectra of mutations are comparable, and include nonsense mutations, small deletions/insertions introducing a frameshift as well as missense mutations affecting highly-conserved amino-acids in the protein (Figure 5), which would probably cause loss-of-function of the mutated allele. Messenger RNAs with mutations introducing premature termination codons (PTC) have indeed been shown to be degraded via the nonsense-mediated mRNA decay (NMD) surveillance system of the cell in fibroblasts from EFMR patients [6]. The identification of a whole gene deletion in the mosaic male patient with PCDH19-DS also supports the loss-of-function as the main consequence of the mutations. However, all the point mutations identified so far are clustered in the large exon 1 of the gene corresponding to the extra-cellular cadherin domain of the protocadherin 19 protein, as previously reported by Dibbens and collaborators [6]. Further studies are needed to determine whether PTC mutations can be found in other exons; this would be expected if the loss-of-function assumption is correct.

EFMR and PCDH19-DS are paradoxical X-linked disorders in which mutated females are severely affected whereas males carrying the mutation are phenotypically unaffected: they have normal cognitive function and no seizures although a subtle psychiatric carrier status was evoked [6],[23]. All affected patients with point mutations identified in this study were also females. In families 2, 4, 6, 8 and 12, the mutation was inherited from the father. Five males (families 2, 6 and 8) were asymptomatic carriers of *PCDH19* mutations, they were healthy, had no cognitive impairment or epilepsy, and none had histories of febrile seizures. In family 4, however, the father who transmitted the mutation to his daughter had moderate mental retardation but no epilepsy. The link between the mutation and his cognitive impairment remains, however, uncertain. The only definitely affected male was, therefore, the patient who was mosaic for the *PCDH19* deletion. There was no molecular evidence of mosaicism in the blood of the father in family 4.

**Figure 6:**
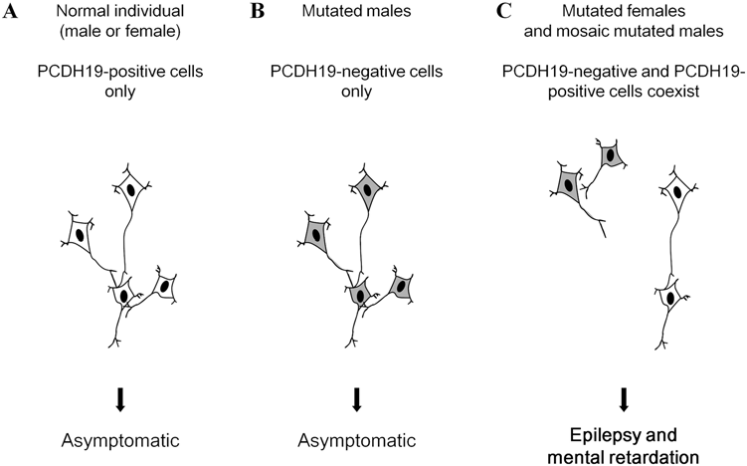
Schematic illustration of the cellular interference mechanism associated with *PCDH19* mutations. A) In normal individuals, characterized by a homogeneous population of *PCDH19*-positive cells, neurons are able to form normal neuronal networks; B) In mutated male patients, hemizygosity leads to a homogeneous population of *PCDH19*-negative cells; in this condition, neurons preserve the ability to form normal neuronal networks; C) In heterozygous mutated females, random X inactivation leads to the co-existence of two *PCDH19*-positive and *PCDH19*-negative cell populations. These two cell populations cause divergent cell sorting and migration (due to attractive or repulsive interactions) and lead to abnormal neuronal networks. Somatic mosaicism in mutated males gives rise to the same pathological situation. The precise mechanisms by which the neuronal networks are altered are still unknown.

Several mechanisms have been suggested to account for the unusual mode of inheritance observed in EFMR. 1) A dominant negative effect of the mutant protein in females (as for mutations in *STK9*/*CDKL5* and *MECP2*) is unlikely, since it is usually associated with lethality in males. 2) Compensatory factors may exist in males; in particular, a protocadherin gene on the Y chromosome (*PCDH11Y*) is specifically expressed in males and could play a role in a sex-dependent compensation; a paralogous gene is located on the X chromosome (*PCDH11X*), but the proteins encoded by the two genes are not identical [6]. In addition, the protocadherin family contains more than 80 genes scattered throughout the human genome [24], supporting the hypothesis of molecular compensation. 3) Another explanation for the unusual mode of inheritance associated with *PCDH19* mutations is cellular interference, a mechanism reminiscent of metabolic interference [6],[21],[25]. It postulates that random inactivation of one X chromosome in mutated females generates tissue mosaicism (i.e. co-existence of *PCDH19*-positive or *PCDH19*-negative cells), which would be pathogenic by altering cell-cell interactions; normal individuals and mutated males, who are homogeneous for *PCDH19*-positive or *PCDH19*-negative cells respectively, would not develop the disease (Figure 6). The identification of an affected male who was mosaic for the *PCDH19* deletion in his fibroblasts, and therefore had *PCDH19*-positive and *PCDH19*-negative cells in this tissue, strongly supports the hypothesis of cellular interference as the main pathogenic mechanism associated with *PCDH19* mutations. The co-existence of normal and mutated cells and the proportion of each population in the brain of this patient cannot, however, be extrapolated from fibroblasts or lymphocytes. To definitely establish that cellular interference is the pathogenic mechanism, it is necessary i) to demonstrate that neuronal cells are mosaic, but also that ii) females who are homozygous for *PCDH19* mutations or deletions are also unaffected, like hemizygous males. Although pathogenesis in cells that express the mutated allele after inactivation corresponds to a loss-of-function, cellular interference would result in a gain-of-function at the tissue level, because of abnormal interactions between mutated and normal cells. This hypothesis supposes that the loss of protocadherin 19 is compensated for, but by a mechanism that is relatively independent of gender.

The same X-linked pattern of inheritance and has been observed for craniofrontonasal syndrome (CFNS), a disorder in which females have multiple skeletal malformations. The gene responsible for CFNS is EFNB1, located in Xq12 and encoding Ephrin B1, a transmembrane protein that is a ligand for Eph receptors [21]. The Ephrin B1/Eph interaction plays a role in cell migration and pattern formation during developmental morphogenesis [26]. Cellular interference, also proposed as the pathogenic mechanism for CFNS [21], had previously been demonstrated in female mice heterozygous for Ephrin B [27]. Although homozygous female and hemizygous male mice showed comparable perinatal lethality due to major skeletal abnormalities, heterozygous females were even more affected, and they alone had polydactyly. Ephrin B1-EphB receptor signaling was shown to regulate skeletal development by controlling cell movement. Mosaic expression of Ephrin B1, caused by random X inactivation in heterozygous females, results in ectopic interactions between the Ephrin B1 ligand and EphB receptors, sufficient to induce the skeletal defects [27].

Protocadherin 19 is an 1148 amino-acids transmembrane protein belonging to the protocadherin delta2 subclass of the cadherin superfamily, which is highly expressed in neural tissues and at different developmental stages [6],[28],[29]. The precise functions of the protein remain so far unknown. However, Delta protocadherins were reported to mediate cell-cell adhesion in vitro and cell sorting in vivo, and could regulate the establishment of neuronal connections during brain development [24],[30]. Ephrin B1 and protocadherin 19 could therefore share major characteristics. Several isoforms of protocadherin 19 have been reported to result from alternative splicing of exon 2 and the existence of two acceptor sites for intron 4 which adds a residue at the beginning of exon 5. The isoform(s) implicated in the physiopathology of EFMR and PCDH19-DS are still not known. Functional studies as well as the development of mouse models are now needed to confirm and unravel the molecular mechanisms of cellular interference in these diseases.

In conclusion, these results extend the clinical spectrum associated with *PCDH19* mutations: we demonstrated that mutations in this gene are not limited to familial female patients, but can also account for isolated cases. Our results suggest that isolated mosaic male patients are also susceptible to the disease. Finally, mutations in *PCDH19* can cause an early and severe epileptic encephalopathy mimicking DS, a major problem for differential diagnosis. The high frequency of patients with *PCDH19* found in this study justifies the molecular testing of this gene in *SCN1A*-negative patients, especially females, diagnosed as having Dravet syndrome. This study also validates the use of SNP microarrays to identify novel genes in isolated patients with severe genetic pathologies. This strategy will hopefully identify new genomic regions or genes that would account for the ∼15–20% of DS patients that do not have *SCN1A* and *PCDH19* mutations.

## Materials and Methods

### Patients

A total of in 74 probands (45 females and 29 males), referred by specialized neuropediatric centres as having Dravet syndrome but who were negative for point mutations or rearrangements in *SCN1A*, were included in the study [15]. Forty-one of these patients were initially selected for the microarray analysis and 33 were later on included for sequencing of *PCDH19*. The referring physicians filled out detailed clinical questionnaires for every patient. Clinical histories were also obtained when possible to assess the evolution of the disease. All clinical reports and questionnaires were re-examined by the same neuropediatrician (RN). Intellectual assessment was based on psychological evaluation when available. Psychomotor skills and cognitive delay were clinically evaluated in all patients. The clinical diagnosis of DS included: normal cognitive and motor development prior to seizures onset, onset of the seizures before the age of one year, seizures mainly triggered by fever, long-lasting seizures (>15 min, that might evolve to *status epilepticus*), later occurrence of other types of seizures (febrile and afebrile) and cognitive regression. The presence of myoclonic jerks and/or ataxia was considered to be a highly characteristic, although inconstant, feature of the disease that could reinforce a diagnosis; however, their absence did not exclude the clinical diagnosis of Dravet syndrome, since they were not previously observed in all patients with DS [7],[31]. Informed written consent was obtained from the patients' parents before blood sampling. This study was approved by the ethical committee (CCPPRB of Pitié-Salpêtrière Hospital, Paris, n°69-03, 25/9/2003).

### Screening for Genomic Rearrangements with High-Density SNP Arrays

Patients were screened using Illumina 370CNV-Duo genotyping BeadChip arrays (370 K). The Infinium II Genotyping reaction steps were performed according to the manufacturer's specifications (Illumina, San Diego, CA) on the P3S platform (Pitié-Salpêtrière Hospital). Briefly, 750 ng of genomic DNA were isothermally amplified at 37°C overnight. The amplified products were fragmented by a controlled enzymatic process then precipitated with isopropanol. The dried precipitated pellet was resuspended, hybridized to 370CNV-Duo beadchips in a capillary flow-through chamber and incubated overnight at 48°C. The amplified, fragmented DNA samples anneal to locus-specific 50-mers during the hybridization step. Each bead type corresponds to one allele per SNP locus. After hybridization, allelic specificity was conferred by enzymatic single-base extension and fluorescent staining. Arrays were washed and dried for 1 h before imaging using a BeadArray Reader (Illumina). Image data analysis and automated genotype calling was performed using Beadstudio 3.1 (Illumina). All genomic positions were based on the UCSC and Ensembl Genome Browsers. Each copy number variant (CNV) identified in patients was searched in the database of genomic variants (http://projects.tcag.ca/variation/), which repertories the structural variation in the Human genome, to determine whether this CNV is normally present in a control population.

### Analysis of the Xq22.1 Deletion with Nimblegen CGH Arrays

Genomic DNA from the patients was analysed by microarray-based comparative genomic hybridization with the HG18 WG Tiling 385 K CGH array v2.0 (Roche NimbleGen, Madison, WI), according to the NimbleGen hybridization Kit Protocol. Briefly, DNA samples from patients and controls were labelled by random priming: the DNA (1 µg) was denatured in the presence of 5′Cy3- or Cy5-labeled random nanomers (Trilink Biotehcnologies, San Diego, CA) and incubated with 100 units of exo-klenow fragment (NEB, Beverly, MA) and dNTP mix [6 mM each in TE buffer (10 mMTris/1 mM EDTA, pH 7.4, Invitrogen)] for 2 h at 37°C. Reactions were terminated by addition of 0.5 mM EDTA (pH 8.0), precipitated with isopropanol and resuspended in water. The Cy-labelled test sample (Cy3) and the reference sample (Cy5) were combined in 13 µL of Nimblegen Hybridization solution (Roche Nimblegen). After denaturation, hybridization was carried out on a MAUI Hybridization System (BioMicro Systems, Salt Lake City, NE) for 18 h at 42°C. The array was washed with the NimbleGen Wash System (Roche NimbleGen), dried by centrifugation and scanned with the genePix 4000B scanner (Axon Instrument, Union City, CA). Fluorescence intensity (raw data) was obtained from the scanned images of the oligonucleotide tiling arrays with NIMBLESCAN 2.0 extraction software (Nimblegen Systems). For each spot on the array, log2 ratios of the Cy3-labeled test sample versus Cy5 reference sample were calculated. Regions were considered to be duplicated or deleted when result exceeded the +/−0.25.

### Sequencing of the PCDH19 Coding Sequence

Eleven specific primer pairs were designed to amplify the 6 exons and adjacent intron-exon boundaries (∼100 bp from each side of the exons) of the *PCDH19* gene (transcript reference EF676096). Primer sequences are available on request. Forward and reverse sequence reactions were performed with the Big Dye Terminator Cycle Sequencing Ready Reaction Kit (PE Applied Biosystems) using the same primers. G50-purified sequence products were run on an ABI 3730 automated sequencer (PE Applied Biosystems) and data were analyzed with the Seqscape 2.5 software (Applied Biosystems). Mutations identified in the patients were looked for directly in the DNA of available parents by sequencing the corresponding amplicon. If neither parent had the mutation, the parents were tested with microsatellite markers at the Xq22.1 locus to ensure that the mutation occurred de novo. In addition, 180 European controls (90 males and 90 females) were included to test new variants in the *PCDH19* gene.

### Fluorescence In Situ Hybridization (FISH)

FISH experiments were performed on peripheral blood lymphocytes (blood samples) and fibroblasts (skin biopsies). Fibroblasts were grown in Dulbecco's modified Eagle's medium containing 4.5 mg/ml glucose and 110 µg/ml pyruvate (DMEM) supplemented with 10% fetal calf serum (FCS), 0.03% glutamine, 1000 U/ml penicillin/streptomycin in a 5% CO_2_ atmosphere for 2 weeks before FISH. Lymphocytes were grown in PB-Max medium (Invitrogen) for 3 days. Metaphase chromosome spreads were obtained by standard hypotonic treatment and methanol/acetate (3/1) fixation. The slides were washed with the cytology FISH accessory kit (Dako). A FISH DNA probe, specific for the Xq22.1 region covering *PCDH19*, was labeled with rhodamine by nick-translation after amplification of the RP11-99E24 BAC (Invitrogen) and cohybridized with a commercial subtelomeric control probe (Cytocell), specific for the pseudo-autosomal region 1 (chromosomes X/Y) labeled with fluorescein isothiocyanate (FITC). The slides were then washed and counterstained with 4,6-diamino-2-phenylindole (DAPI) for chromosome identification. Metaphase cells were examined under a motorized reflected BX61 Olympus fluorescence microscope with filters for separate detection of DAPI, FITC and rhodamine. One hundred metaphase cells were counted to determine the degree of mosaicism in fibroblasts and lymphocytes. Metaphase chromosomes from a karyotypically normal female were used as a control.

### Statistical Tests

Frequencies were compared with the Chi-Square test or the Fisher exact test when appropriate. Means were compared using Mann-Whitney Rank Sum Test. Statistical analysis was performed using SigmaStat 3.5 software.
