# Correction: Sporadic Infantile Epileptic Encephalopathy Caused by Mutations in *PCDH19* Resembles Dravet Syndrome but Mainly Affects Females

**DOI:** 10.1371/annotation/314060d5-06da-46e0-b9e4-57194e8ece3a

**Published:** 2009-04-03

**Authors:** Christel Depienne, Delphine Bouteiller, Boris Keren, Emmanuel Cheuret, Karine Poirier, Oriane Trouillard, Baya Benyahia, Chloé Quelin, Wassila Carpentier, Sophie Julia, Alexandra Afenjar, Agnès Gautier, François Rivier, Sophie Meyer, Patrick Berquin, Marie Hélias, Isabelle Py, Serge Rivera, Nadia Bahi-Buisson, Isabelle Gourfinkel-An, Cécile Cazeneuve, Merle Ruberg, Alexis Brice, Rima Nabbout, Eric LeGuern

The affiliations for the 20th author, Isabelle Gourfinkel-An, and the 24th author, Rima Nabbout, were incorrect. Dr. Gourfinkel-An is affiliated with: INSERM U975 (Ex-U679), Paris, France; Plate-forme Post-Génomique P3S, UPMC, Faculté de Médecine, Paris, France; and Centre de Référence Épilepsies Rares, Paris, France. Dr. Nabbout is affiliated with: Département de Neuropédiatrie, AP-HP, Hôpital Necker-Enfants Malades, Paris-Descartes, Paris, France; and Centre de Référence Épilepsies Rares, Paris, France.

